# Age-Related Changes in Standing Balance in Preschoolers Using Traditional and Nonlinear Methods

**DOI:** 10.3389/fphys.2021.625553

**Published:** 2021-02-22

**Authors:** Zengming Hao, Yi Yang, Anke Hua, Ying Gao, Jian Wang

**Affiliations:** ^1^Department of Sports Science, College of Education, Zhejiang University, Hangzhou, China; ^2^Center for Psychological Sciences, Zhejiang University, Hangzhou, China

**Keywords:** standing balance, preschool children, nonlinear analysis, recurrence quantification analysis, detrended fluctuation analysis

## Abstract

Considerable disagreement exists on the linearity of the development of standing balance in children. This study aimed to use different traditional and nonlinear methods to investigate age-related changes in standing balance in preschoolers. A sample of 118 preschoolers took part in this study. A force platform was used to record the center of pressure during standing balance over 15 s in three conditions: eyes open, eyes closed, and/or head extended backward. Detrended fluctuation analysis (DFA), recurrence quantification analysis (RQA), and traditional measures were used to evaluate standing balance. The main results are as follows: (1) Higher range and SD in the anterior-posterior (AP) direction were observed for 5-year-old than for 4-year-old children, while higher DFA coefficient (at shorter time scales) and higher determinism and laminarity in the AP direction were found for 5-year-old children compared to 3- and 4-year-old children; and (2) as sensory conditions became more challenging, all traditional measures increased and DFA coefficients (at shorter and longer time scales) decreased in the AP and mediolateral directions, while determinism and laminarity significantly declined in the AP direction. In conclusion, although increased postural sway, 5-year-old preschool children’s balance performance improved, and their control strategy changed significantly compared with the younger preschoolers. Sensory perturbation (eye closure and/or head extension) changed preschoolers’ balance performance and control strategy. Moreover, both traditional and nonlinear methods provided complementary information on the control of standing balance in preschoolers.

## Introduction

Maintaining standing balance is a complex sensorimotor process. It involves multiple sensory systems and actions of muscles distributed over the whole body. A deficit in any sensory systems or integration of multisensory information can affect standing balance (Balasubramaniam and Wing, 2002; Molloy et al., 2003). The control of standing balance is affected by perceptual information, attention, and cognitive processes (Balasubramaniam and Wing, 2002). It should be noted that age is an essential factor affecting standing balance (Hsu et al., 2009). Several studies have found that the ability to control standing balance develops during childhood until early adult life and deteriorates from 40 to 59 years (Sheldon, 1963; Goble and Baweja, 2018). However, these studies did not cover preschool years, and the development of sensory systems and central nervous systems integration for preschool children is incomplete (Steindl et al., 2006; Hsu et al., 2009; Sá et al., 2018). Nonetheless, intra-modal reweighting was exhibited in children as young as 4 years of age, while inter-modal reweighting was only observed in older children (Bair et al., 2007; Rinaldi et al., 2009). Preschoolers may not effectively suppress the influence of unreliable proprioception and visual information on standing balance (Forssberg and Nashner, 1982; Foudriat et al., 1993). Especially for 3-year-old children, many of them fail to maintain standing balance under some challenging conditions (e.g., eyes closed and/or on a foam surface; Slobounov and Newell, 1994; Verbecque et al., 2016a). Several studies found that younger children sway more with the eyes open than with the eyes closed (Riach and Hayes, 1987; Slobounov and Newell, 1994; Newell et al., 1997a,b). Moreover, an increase in sway amplitude was found for 5-year-old children compared with 3- and 4-year-old children in another study (Verbecque et al., 2016a). These phenomena conflict with the traditional view of the influence of vision and age on standing balance. For typically developing children, postural sway decreases with increasing age under different sensory conditions (Sá et al., 2018; Villarrasa-Sapiña et al., 2019). Nonetheless, considerable disagreement exists on whether this developmental trend occurs linearly or whether turning points can be identified (Kirshenbaum et al., 2001; Rival et al., 2005; Verbecque et al., 2016b).

The most common method for assessing standing balance is the postural sway’s characterization by measuring the center of pressure (COP) displacements (Verbecque et al., 2016a). COP signals can be used as an effective method to determine whether a child has sufficient postural control under different sensory conditions. Several studies have shown that COP signals are non-random and nonstationary, containing structural information of the postural control system (Collins and De Luca, 1993; van den Hoorn et al., 2018). However, traditional methods (e.g., range, SD, root mean square, sway velocity, sway path length, and sway area) have been usually used to evaluate the COP signals by assuming that postural sway is stationary (Verbecque et al., 2016a,b). In fact, traditional methods have some limitations for assessing standing balance. For example, one study found that the sway area did not distinguish standing balance between 5-year-old children and 3-year-old children (Slobounov and Newell, 1994). Another study’s results showed that sway velocity and Romberg quotient of most traditional measures remained unaltered among different age groups of preschool children (Verbecque et al., 2016a). These results indicate that conventional methods may ignore some critical information about standing balance. In contrast, many nonlinear methods are based on concepts of chaos, fractals, and complexity (Ma et al., 2018; Henriques et al., 2020), which have been used to evaluate the COP signals to understand the dynamics of standing balance in different groups (Doyle et al., 2005; Seigle et al., 2009; Ramdani et al., 2013; Rigoldi et al., 2014; Zhou et al., 2017; Lobo Da Costa et al., 2019). Postural sway variability can be quantified using multiscale entropy (MSE) and fractal dimension (FD). Older adults with lower postural sway complexity experienced more falls in the future, while traditional measures were not associated with future falls (Zhou et al., 2017). FD measures are more reliable than traditional COP measures in assessing standing balance (Doyle et al., 2005). Detrended fluctuation analysis (DFA) can assess the persistent and anti-persistent behaviors of COP signals in different time scales (Peng et al., 1995; Teresa Blázquez et al., 2009, 2010). Besides, recurrence quantification analysis (RQA) was used to investigate the dynamical properties of COP signals, even for a short duration and for nonstationary data (Sylos Labini et al., 2012). Some studies have shown that RQA measures in the anterior-posterior direction are sufficient to distinguish the young and elderly group and even distinguish non-fallers and fallers (Seigle et al., 2009; van den Hoorn et al., 2018). Thus, using nonlinear methods may provide crucial information about COP signals, contributing to more accurate insights into standing balance.

In previous studies, most authors investigated the age-related changes in standing balance in preschoolers under challenging conditions by perturbating the sensory inputs of vision and/or proprioception (Verbecque et al., 2016a,b). Especially for the condition of standing on a foam surface with eyes closed, the vestibular input dominated because both visual and somatosensory inputs had been removed or reduced (Young, 2015). In contrast, preschoolers’ development of the vestibular system did not reach functional maturity (Steindl et al., 2006; Hsu et al., 2009; Sá et al., 2018). During the preschool period, proprioception may be the only relatively reliable sensory input for standing balance (Steindl et al., 2006; Hsu et al., 2009). The head extension is also an effective method for evaluating standing balance (Kogler et al., 2000; Buckley et al., 2005; Vuillerme and Rougier, 2005; Paloski et al., 2006; Vuillerme et al., 2008). The head-extended posture is recognized to induce a modification of the vestibular inputs and abnormal sensory inputs from neck proprioceptors, representing a challenge for the postural control system (Vuillerme et al., 2008). Most previous studies reported a standing duration of 30 s and above (Verbecque et al., 2016b). Choosing a longer time for data recording has the advantage of being a more realistic estimation of the standing balance of preschool children. However, it is difficult for children below 5 years to maintain balance with eyes closed for longer durations (Forssberg and Nashner, 1982; Verbecque et al., 2016a). Moreover, preschoolers are easily distracted (Lobo Da Costa et al., 2019). Postural sway data of 15 or 20 s can effectively distinguish the standing balance among different age groups (Newell et al., 1997a; Goble and Baweja, 2018; van den Hoorn et al., 2018). Therefore, a shorter duration (e.g., 15 s) may be suitable for evaluating preschool children’s standing balance. In general, the longer the time for data recording, the better the reliability of COP signals’ measures. Nonetheless, some nonlinear measures applied to short data also had better reliability (Doyle et al., 2005; Teresa Blázquez et al., 2009; Sylos Labini et al., 2012; van den Hoorn et al., 2018). Fractal measures and RQA measures are more reliable than traditional measures of COP signals in assessing standing balance for a duration of 10 or 15 s (Doyle et al., 2005; van den Hoorn et al., 2018).

Thus, the purpose of the current study was to investigate how age and sensory perturbation affect the control of standing balance for preschool children on a firm surface using traditional and nonlinear methods. The hypotheses were as follows: (1) The balance performance and control strategy of 5-year-old preschool children’s standing balance would change significantly compared with the younger preschoolers; (2) sensory perturbations (eye closure and/or head extension) would change the control of preschoolers’ standing balance accordingly; and (3) both traditional and nonlinear methods may discriminate the age-related changes in standing balance in preschoolers, and nonlinear methods may provide different information about the effect of age on standing balance.

## Materials and Methods

### Participants

A cross-sectional study was performed in a sample of 118 preschool children. They were grouped according to chronological age: 3-year-old children (*n* = 40), 4-year-old children (*n* = 39), and 5-year-old children (*n* = 39). The parents or guardians of children provided written consent before any measurements. A questionnaire was also completed by the parents or guardians to identify any presence among participants of developmental problems or interest in cooperation, which were all considered exclusion criteria. Participants were recruited from one of the regular preschools in Hangzhou, China. This study was approved by the local ethical committee of Zhejiang University (issued no. 2020-003) and conformed to the Declaration of Helsinki.

Among the 3-year-old children, 90% completed all conditions (four children were excluded because of test failure). Among the 4-year-old children, 97.44% completed all conditions (only one child was excluded because of test failure). Among the 5-year-old children, 100% were able to complete all conditions. In total, the final sample of 113 children are presented, which include 3-year-old children (*n* = 36), 4-year-old children (*n* = 38), and 5-year-old children (*n* = 39). Table 1 presents the descriptive characteristics of the participants. Body height and body mass increase significantly with age (*p* < 0.01 for height and body mass).

**Table 1:** Descriptive characteristics of the participants.

Age group	P	N	Gender (M/F)	Height (cm)	Body mass (kg)
3 years	90%	36	19/17	103.44 ± 4.96	17.35 ± 3.16
4 years	97.44%	38	17/21	111.53 ± 4.03	19.19 ± 3.30
5 years	100%	39	21/18	119.71 ± 6.61	23.38 ± 5.47

P, percentage of children that completed all three conditions; N, number of children of whom the results were analyzed; M, male; F, female.

### Data Collection

In an upright bipedal stance, participants were asked to stand barefoot on a force plate (0.4 × 0.5 m, 1,000 Hz, model OR 6-5-2000, AMTI Inc., United States) with feet together (Verbecque et al., 2016b) for 15 s. They were asked to stand on a firm surface and keep their arms beside their bodies and stand as still as possible. Standing balance was measured in three non-randomized test conditions: (1) EO: eyes open; (2) EC: eyes closed; and (3) ECHB: eyes closed and head extended backward (Smith et al., 2012). Participants were asked to keep their head in a straight-ahead direction under the condition of EO and EC, and they were asked to tilt their head backward for at least 45° under the condition of ECHB. Each condition was designed to remove or reduce sensory inputs. For the condition of EO, all sensory inputs are available; for the condition of EC, only the visual information is unavailable; and for the condition of ECHB, sensory inputs arising from vision, vestibular system, and neck proprioceptors are removed or reduced (Vuillerme et al., 2008; Smith et al., 2012). All participants familiarize themselves with each condition before the formal test and have 30 s of rest between different conditions. According to previous studies, a visual target used in the condition of EO can enhance children’s attention and motivation (Schärli et al., 2013; Verbecque et al., 2016b), and gazing at objects at a near distance (small eye–object distance) can reduce body sway (Verbecque et al., 2016b; Aoki et al., 2018). Thus, in the EO condition, the children were instructed to look at a stationary marker positioned 1 m away and individually adjusted for the eye height. One investigator stayed close to the participant throughout the entire test to prevent them from falling. Once the participant moved their feet or fell, the trial was stopped, and the results were excluded for further analysis.

### Data Analysis

Demographic data (gender, height, and body mass) were reported. All signals from the force platform were processed offline using MATLAB software (MathWorks, Natick, MA, United States). The COP positions were calculated from the ground reaction forces and moments of force and then filtered using a 20 Hz low-pass, 2nd order, zero-lag Butterworth filter. Furthermore, the mean of the filtered data was removed. The COP displacements were subsequently analyzed using traditional and nonlinear methods in the anterior-posterior (AP) and mediolateral (ML) directions. Traditional methods included the range, SD, sway mean velocity, sway path length, and sway area, which quantified the postural sway (Verbecque et al., 2016a,b). Nonlinear methods included the DFA and RQA.

The range is the distance between the maximum and minimum COP displacement in the AP and ML directions, representing the entire trial’s postural sway. In general, the greater the range, the worse the postural stability (Palmieri et al., 2002; Paillard and Noé, 2015). Because the COP signal of zero mean, SD, and root mean square (RMS) provide the same result, which is defined as the square root of the mean of the squares of COP displacement in the AP and ML directions. SD is a variability index of COP displacements (Palmieri et al., 2002; Paillard and Noé, 2015; Luo et al., 2018). Path length quantifies the magnitude of the two-dimensional displacement based on the total distance traveled and is considered a valid index (the smaller the path length, the better the postural stability; Paillard and Noé, 2015). Sway mean velocity is calculated by dividing the COP excursion by the duration time, which is considered an index with the greatest reliability, reflecting the efficiency of postural control (the smaller the velocity, the better the postural control; Paillard and Noé, 2015; Luo et al., 2018; van den Hoorn et al., 2018). We calculated Sway mean velocity and Path length of the COP signal as follows:

$$MV\_ml=\sum_{i=1}^{N}\left|x\left(i+1\right)-x\left(i\right)\right|*F/N\;$$

$$MV\_ap=\sum_{i=1}^{N}\left|y\left(i+1\right)-y\left(i\right)\right|*F/N\;$$

$$Path=\sum_{i=1}^{N}\sqrt{{\left(x\left(i+1\right)-x\left(i\right)\right)}^{2}+{\left(y\left(i+1\right)-y\left(i\right)\right)}^{2}}\;$$

where x(i) and y(i) are the COP displacements in the ML and AP directions, respectively, *N* = number of samples, F = sampling frequency.

**Figure 1:**
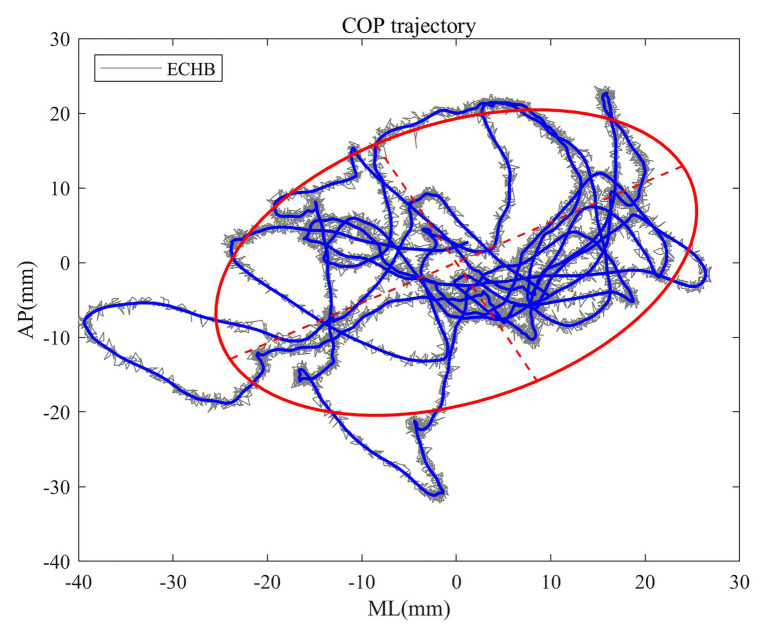
The raw (gray trace) and filtered (blue trace) center of pressure (COP) signals for a 4-year-old child under the condition of eyes closed and head extended backward (ECHB) during 15 s. Estimated sway area (red trace) is superimposed onto the plots of COP trajectory.

Sway area quantifies 85% of the total area covered in the ML and AP directions using an ellipse to fit the COP data, which is considered an index of overall postural performance (Paillard and Noé, 2015; Verbecque et al., 2016a). Figure 1 shows some details of the sway area calculation of COP trajectory from a 4-year-old child under the condition of ECHB. It can be seen that all traditional measures of COP signals reflect the balance performance with the small the value, the better the balance performance.

**Figure 2:**
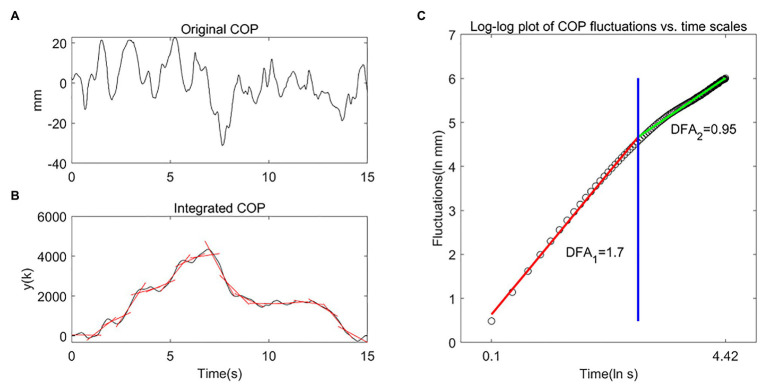
Detrended fluctuation analysis (DFA) method and DFA coefficients. **(A)** Original COP signal in the anterior-posterior (AP) direction from a 4-year-old child under the condition of ECHB during 15 s. **(B)** Integrated COP signal y(k), the red solid line represent the linear trend in each box with 50% overlap. **(C)** Log-log plot of COP fluctuations vs. time scales, DFA_1_ (red line) and DFA_2_ (green line) reflect the COP fluctuations at shorter (to the left of the blue line) and longer (to the right of the blue line) time scales, respectively.

Detrended fluctuation analysis is a technique for quantifying the long-range correlation behavior in a time series (Peng et al., 1995), and frequently used to study the behavior of the COP trajectory (Teresa Blázquez et al., 2009, 2010; van den Hoorn et al., 2018; Lobo Da Costa et al., 2019). DFA can measure the relation between COP fluctuations at different time scales by the slope of a linear region on the log-log plot of COP fluctuations vs. time scales (Figure 2). Based on the shorter duration of tests in this study, the time window ranged from 0.10 to 4.42 s (van den Hoorn et al., 2018). The COP signal was integrated over time and divided into smaller time windows with 50% overlap. Each time window’s linear trend was subtracted, and the root-mean-square fluctuations of the integrated COP around the linear fits were determined. For the log-log plot of COP fluctuations vs. time scales, two linear regions were fitted by minimizing the squared errors between the two fitted lines and actual data (Figure 2), and two slopes of different time scales were determined. The first slope (DFA_1_) in general greater than 1.5, indicating that a persistent pattern of COP sway. The second slope (DFA_2_) in general smaller than 1.5, indicating that an anti-persistent pattern of COP sway. Higher DFA values indicate smoother and more persistent behavior at short (DFA_1_) and long (DFA_2_) time scales, while lower DFA values indicate less smooth and more anti-persistent behavior at short (DFA_1_) and long (DFA_2_) time scales (van den Hoorn et al., 2018).

We calculated DFA_1_ and DFA_2_ of the COP signal as follows:

The original COP signal is integrated as: $y\left(k\right)=\overset{k}{\underset{t=1}{\sum }}\left[x\left(t\right)-\overset{-}{x}\right]$

where *x*(t) is the original COP signal at time *t*, $\overset{-}{x}$ is the average of the entire time series, and $y\left(k\right)$ is the integrated COP signal.

The fluctuation of the integrated COP signal is calculated as: $F\left(n\right)=\sqrt{\frac{1}{N}\overset{N}{\underset{k=1}{\sum }}{\left[y\left(k\right)-y_{n}\left(k\right)\right]}^{2}}$

where $y_{n}\left(k\right)$ is the local linear trend, $F\left(n\right)$ will increase with the box size *n*. The slopes of the fitted lines of the log-log plot at short and long time scales are the DFA coefficients (DFA_1_ and DFA_2_).

**Figure 3:**
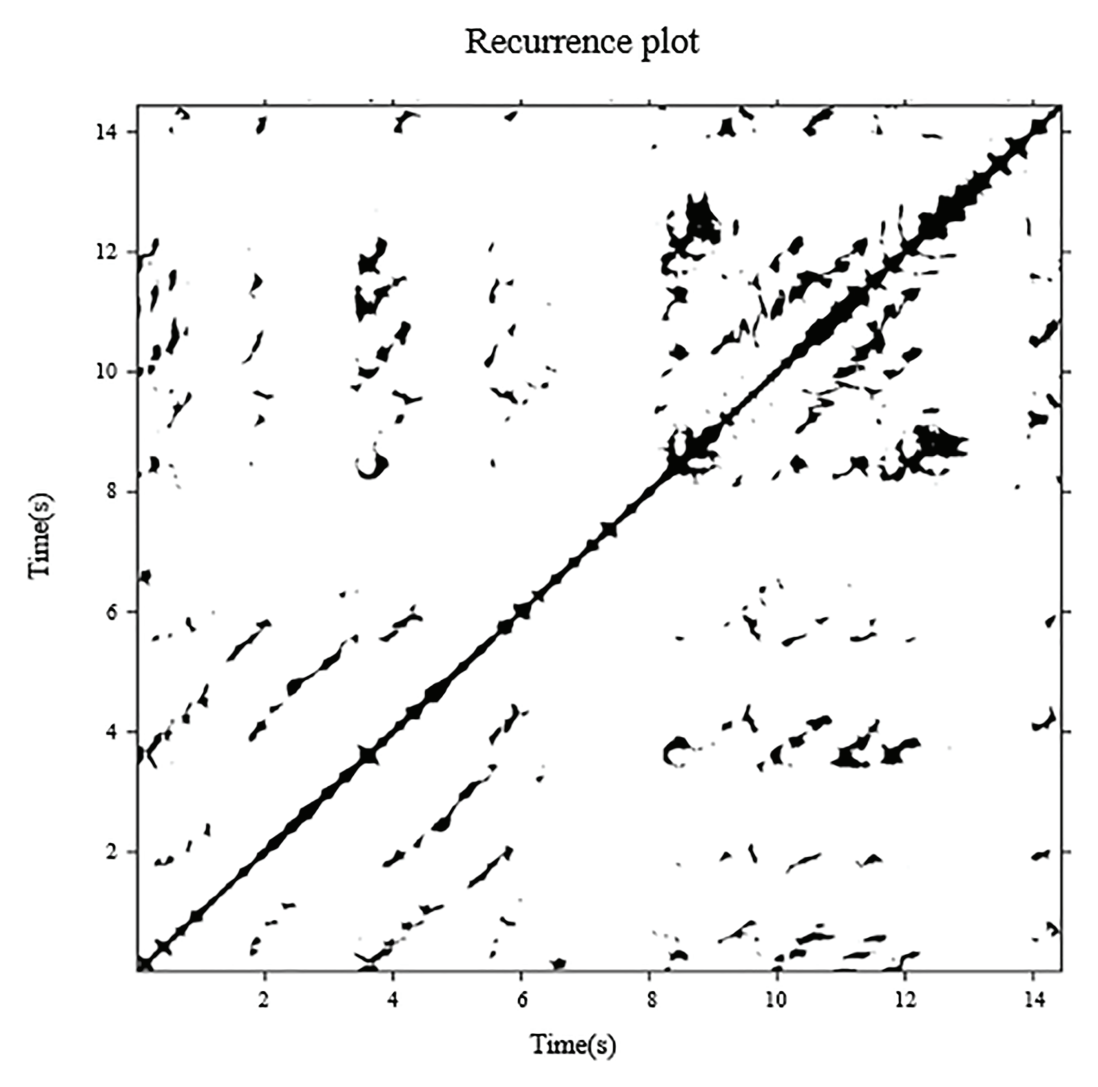
Recurrence plot of a COP signal in the AP direction from a 4-year-old child under the condition of ECHB duration 15 s (time delay = 28 samples, embedded dimension = 3, and recurrence rate = 5%).

Recurrence quantification analysis is a tool for studying the dynamics of a signal. It is based on the construction of a recurrence plot (RP) from which quantitative measures are extracted. The time delay was calculated using the mutual information method, and the embedded dimension was determined using false nearest neighbor analysis. Figure 3 shows a recurrence plot of a COP signal in the AP direction from a 4-year-old child under the condition of ECHB. RP’s features can be quantified by the diagonal lines and vertical lines using Marwan’s RQA toolbox (Marwan et al., 2007). Determinism (%DET) refers to the percentage of all recurrences in phase space that form diagonal line lengths longer than a pre-set threshold distance. Higher %DET values indicate a more predictable, less random COP data motion, which is consistent with better balance performance (van den Hoorn et al., 2018). Laminarity (%LAM) refers to the percentage of all recurrences in phase space that forms vertical line lengths longer than a pre-set threshold distance. Higher %LAM values indicate a more intermittent COP motion with more periods of minimal COP fluctuations (van den Hoorn et al., 2018). To avoid the ceiling effect of the %DET and %LAM, minimal length of both diagonal and vertical line features were set as 0.1 s; the recurrence threshold was chosen as 5% of the recurrence rate (Seigle et al., 2009; Ramdani et al., 2013; van den Hoorn et al., 2018).

We calculated Determinism (%DET) and Laminarity (%LAM) of the COP signal as follows:

Recurrence plot is briefly defined as $R_{i,j}^{m,\varepsilon_{i}}=\Theta \left(\varepsilon_{i}-\left\|{\vec{x}}_{i}\right.-\left.{\vec{x}}_{j}\right\|\right)$

where *ε* is a predefined threshold and ${\vec{x}}_{i}{\vec{x}}_{j}$ are phase space trajectories in an *m*-dimension phase space;

$$\%\mathrm{DET}=\frac{\sum_{l=l_{\min}}^{N}lP^{\varepsilon }\left(l\right)}{\sum_{i,j}^{N}R_{i,j}^{m,\varepsilon }}\times 100$$

where $P^{\varepsilon }\left(l\right)=\left\{l_{i};i=1\ldots N_{l}\right\}\;$ is the frequency distribution of the lengths *l* of diagonal structures, and *N_l_* is the absolute number of diagonal lines;

$$\%\mathrm{LAM}=\frac{\sum_{v=v_{\min}}^{N}vP^{\varepsilon }\left(v\right)}{\sum_{v=1}^{N}vP^{\varepsilon }\left(v\right)}\times 100$$

where $P^{\varepsilon }\left(v\right)=\left\{v_{i};i=1\ldots N_{v}\right\}\;$ denotes the frequency distribution of the lengths *l* of vertical structures.

The following measures were selected for analysis. Traditional measures included amplitude (Range), SD, and sway mean velocity (MV) in the AP and ML directions; sway path length (Path) and sway area (Area) were also included. Nonlinear measures included DFA coefficients (DFA_1_ and DFA_2_), determinism (%DET), and laminarity (%LAM) in the AP and ML directions.

### Statistics

Each measure’s distribution of normality was tested (Shapiro–Wilk test, *p* > 0.05). A mixed repeated-measures ANOVA with between-subject factors (age) and within-subject factors (condition) was conducted to assess the effects of age and condition on all the measures. Significant interactions were explored further using simple effects analyses and performed with Bonferroni *post hoc* tests. Greenhouse-Geisser corrections were applied for circumstances in which sphericity could not be assumed (Mauchly’s test, *p* < 0.05). The significance level was set as *p* < 0.05 with two-tailed. Effect size values (*η*_p_^2^) were reported for ANOVA. All statistical testing was conducted using SPSS (IBM SPSS Statistics, Version 25, SPSS Inc., Chicago, IL, United States).

## Results

### Traditional Measures

Table 2 provides an overview of mean values and SDs for traditional measures of standing balance in each condition classified according to the age group of preschool children. Significant main effects of age were found for Range_ap and SD_ap. *Post hoc* tests revealed that Range_ap (*p* = 0.03) and SD_ap (*p* = 0.03) were significantly higher for the 5-year-old children than for the 4-year-old children. Effect sizes were small to medium, ranging from 0.058 to 0.060 (Table 3). No age-related differences were found for Range_ml, SD_ml, MV_ml, MV_ap, Path, and Area. Significant main effects of condition were observed for all traditional measures. Range_ml, Range_ap, SD_ml, SD_ap, MV_ml, MV_ap, Path, and Area significantly increased as conditions became more challenging (*p* < 0.01, all traditional measures). Effect sizes were large, ranging from 0.354 to 0.600 (Table 4). No significant age by condition interaction effects for Range_ml, Range_ap, SD_ml, SD_ap, MV_ml, MV_ap, Path, and Area.

**Table 2:** Overview of mean values and SDs for traditional measures of standing balance.

	Age group	EO	EC	ECHB
Range_ml (mm)	3 years	33.07 ± 8.85	43.38 ± 12.08	51.26 ± 15.85
	4 years	30.76 ± 8.50	41.53 ± 11.40	52.89 ± 17.31
	5 years	29.69 ± 11.31	44.41 ± 18.57	51.21 ± 17.43
Range_ap (mm)	3 years	32.79 ± 9.35	37.24 ± 11.63	51.95 ± 16.49
	4 years	27.09 ± 7.57	37.27 ± 8.84	47.12 ± 13.43
	5 years	33.92 ± 13.49	42.52 ± 14.94	52.06 ± 19.52
SD_ml (mm)	3 years	6.71 ± 1.64	8.28 ± 2.32	9.98 ± 3.07
	4 years	5.96 ± 1.43	8.12 ± 2.07	9.74 ± 2.85
	5 years	5.77 ± 1.88	8.53 ± 3.25	9.67 ± 2.81
SD_ap (mm)	3 years	6.57 ± 1.85	7.38 ± 2.44	9.78 ± 2.98
	4 years	5.73 ± 1.82	7.26 ± 1.61	9.06 ± 2.33
	5 years	7.17 ± 2.81	8.81 ± 2.97	9.36 ± 2.59
MV_ml (mm/s)	3 years	19.16 ± 6.08	25.39 ± 7.13	31.78 ± 11.36
	4 years	17.53 ± 5.51	26.80 ± 9.19	32.09 ± 11.71
	5 years	17.12 ± 6.27	25.61 ± 11.42	30.88 ± 13.91
MV_ap (mm/s)	3 years	21.85 ± 6.17	26.86 ± 6.95	38.24 ± 10.58
	4 years	17.98 ± 4.24	25.31 ± 7.14	35.22 ± 9.04
	5 years	18.97 ± 8.20	25.71 ± 9.24	35.73 ± 14.87
Path (mm)	3 years	484.76 ± 137.50	617.29 ± 156.29	829.63 ± 243.83
	4 years	420.13 ± 108.33	616.99 ± 182.48	794.92 ± 233.59
	5 years	428.54 ± 169.32	606.14 ± 232.30	788.23 ± 327.28
Area (mm^2^)	3 years	517.25 ± 233.98	755.14 ± 450.46	1213.20 ± 759.88
	4 years	410.59 ± 179.09	718.19 ± 297.22	1082.66 ± 554.14
	5 years	515.29 ± 326.91	936.00 ± 654.67	1111.97 ± 657.01

EO, eyes open; EC, eyes closed; ECHB, eyes closed and head extended backward.

**Table 3:** Main effects of age on traditional measures of standing balance.

	3 years mean	4 years mean	5 years mean	F	*p*	Effect size (η_p_^2^)
Range_ml (mm)	42.57	41.73	41.77	0.079	0.924	0.001
Range_ap (mm)	40.66	37.16^B^	42.83^B^	3.405	**0.037**	0.058
SD_ml (mm)	8.32	7.94	7.99	0.433	0.650	0.008
SD_ap (mm)	7.91	7.35^B^	8.44^B^	3.512	**0.033**	0.060
MV_ml (mm/s)	25.44	25.48	24.54	0.214	0.808	0.004
MV_ap (mm/s)	28.99	26.17	26.81	1.664	0.194	0.029
Path (mm)	643.89	610.68	607.64	0.588	0.557	0.011
Area (mm^2^)	828.53	737.15	854.42	0.965	0.384	0.017

B 4-year-old children ≠ 5-year-old children for *p* < 0.05. Significant differences are shown in bold font.

**Table 4:** Main effects of condition on traditional measures of standing balance.

	EO mean	EC mean	ECHB mean	F	*p*	Effect size (*η*_p_^2^)
Range_ml (mm)	31.17^+^^*^	43.11^+^^#^	51.79^*^^#^	88.703	**<0.001**	0.446
Range_ap (mm)	31.27^+^^*^	39.01^+^^#^	50.38^*^^#^	80.884	**<0.001**	0.424
SD_ml (mm)	6.15^+^^*^	8.31^+^^#^	9.80^*^^#^	108.292	**<0.001**	0.496
SD_ap (mm)	6.49^+^^*^	7.82^+^^#^	9.40^*^^#^	60.311	**<0.001**	0.354
MV_ml (mm/s)	17.94^+^^*^	25.93^+^^#^	31.58^*^^#^	83.950	**<0.001**	0.433
MV_ap (mm/s)	19.60^+^^*^	25.96^+^^#^	36.40^*^^#^	165.037	**<0.001**	0.600
Path (mm)	444.48^+^^*^	613.47^+^^#^	804.27^*^^#^	131.230	**<0.001**	0.544
Area (mm^2^)	481.04^+^^*^	803.11^+^^#^	1135.95^*^^#^	82.315	**<0.001**	0.428

EO, eyes open; EC, eyes closed; ECHB, eyes closed and head extended backwards.

+ EO ≠ EC for *p* < 0.05.

* EO ≠ ECHB for *p* < 0.05.

# EC ≠ ECHB for *p* < 0.05. Significant differences are shown in bold font.

### Nonlinear Measures

Table 5 provides an overview of mean values and SDs for nonlinear measures of standing balance in each condition classified according to the age group of preschool children. Significant main effects of age were found for DFA_1__ap, %DET_ap, and %LAM_ap. *Post hoc* tests revealed that DFA_1__ap, %DET_ap, and %LAM_ap were not statistically different for the 3- and 4-year-old children (*p* > 0.05 for these measures), DFA_1__ap was significantly higher for the 5-year-old children than 3- (*p* < 0.01) and 4-year-old children (*p* = 0.04), %DET_ap was significantly higher for the 5-year-old children than 3- (*p* < 0.01) and 4-year-old children (*p* = 0.02), and %LAM_ap was significantly higher for the 5-year-old children than 3- (*p* < 0.01) and 4-year-old children (*p* < 0.01). Medium to large effect sizes were found for DFA_1__ap, %DET_ap, and %LAM_ap, ranging from 0.093 to 0.153 (Table 6). No age-related differences were found for nonlinear measures (DFA_1__ml, DFA_2__ml, DFA_2__ap, %DET_ml, and %LAM_ml).

**Table 5:** Overview of mean values and SDs for nonlinear measures of standing balance.

	Age group	EO	EC	ECHB
DFA_1__ml	3	1.77 ± 0.04	1.73 ± 0.05	1.70 ± 0.08
	4	1.73 ± 0.06	1.72 ± 0.05	1.70 ± 0.07
	5	1.75 ± 0.05	1.73 ± 0.06	1.71 ± 0.08
DFA_1__ap	3	1.72 ± 0.07	1.65 ± 0.08	1.65 ± 0.09
	4	1.71 ± 0.06	1.67 ± 0.07	1.66 ± 0.08
	5	1.73 ± 0.04	1.71 ± 0.06	1.68 ± 0.08
DFA_2__ml	3	1.22 ± 0.15	0.97 ± 0.25	1.04 ± 0.23
	4	1.08 ± 0.21	0.92 ± 0.23	1.01 ± 0.27
	5	1.07 ± 0.22	1.02 ± 0.23	1.05 ± 0.26
DFA_2__ap	3	1.13 ± 0.20	0.99 ± 0.26	1.00 ± 0.16
	4	1.14 ± 0.20	1.06 ± 0.17	1.04 ± 0.23
	5	1.25 ± 0.20	1.09 ± 0.18	0.98 ± 0.22
%DET_ml	3	80.66 ± 6.46	81.47 ± 4.85	79.20 ± 6.70
	4	80.10 ± 5.78	78.95 ± 6.53	77.98 ± 6.01
	5	79.50 ± 6.55	79.94 ± 5.44	80.21 ± 6.21
%DET_ap	3	72.88 ± 8.51	70.60 ± 7.81	69.31 ± 9.93
	4	75.06 ± 7.03	73.82 ± 7.19	70.17 ± 8.15
	5	79.07 ± 6.62	77.38 ± 7.04	72.89 ± 7.76
%LAM_ml	3	83.36 ± 5.03	82.78 ± 3.87	81.62 ± 5.07
	4	82.89 ± 5.30	80.64 ± 5.94	80.58 ± 4.54
	5	81.73 ± 4.94	82.26 ± 4.39	82.81 ± 5.17
%LAM_ap	3	77.46 ± 6.66	75.76 ± 6.30	74.70 ± 7.75
	4	78.94 ± 5.60	77.93 ± 5.60	74.98 ± 6.81
	5	82.95 ± 5.27	81.19 ± 5.25	76.89 ± 6.31

EO, eyes open; EC, eyes closed; ECHB, eyes closed and head extended backward.

**Table 6:** Main effects of age on nonlinear methods of standing balance.

	3 years mean	4 years mean	5 years mean	F	*p*	Effect size (η_p_^2^)
DFA_1__ml	1.732	1.716	1.732	1.711	0.186	0.031
DFA_1__ap	1.672^A^	1.679^B^	1.706^A^^,^^B^	5.082	**0.008**	0.093
DFA_2__ml	1.075	1.005	1.043	1.858	0.161	0.034
DFA_2__ap	1.042	1.079	1.104	1.968	0.145	0.035
%DET_ml	80.44	79.01	79.88	0.968	0.383	0.017
%DET_ap	70.93^A^	73.01^B^	76.45^A^^,^^B^	9.604	**<0.001**	0.149
%LAM_ml	82.59	81.37	82.26	1.143	0.323	0.020
%LAM_ap	75.97^A^	77.28^B^	80.34^A^^,^^B^	9.906	**<0.001**	0.153

A 3-year-old children ≠ 5-year-old children for *p* < 0.05.

B 4-year-old children ≠ 5-year-old children for *p* < 0.05.Significant differences are shown in bold font.

Significant main effects of condition were found for DFA_1__ml, DFA_1__ap, DFA_2__ml, DFA_2__ap, %DET_ap, and %LAM_ap. *Post hoc* tests revealed that %DET_ap and %LAM_ap (*p* > 0.05) were not statistically different for the EO and EC. %DET_ap was significantly lower for the ECHB than EO (*p* < 0.01) and EC (*p* < 0.01), and %LAM_ap was significantly lower for the ECHB than EO (*p* < 0.01) and EC (*p* < 0.01). DFA_1__ap was significantly lower for the ECHB than EO (*p* < 0.01) and EC (*p* < 0.01), DFA_2__ml was significantly lower for the ECHB than EO (*p* < 0.01) and EC (*p* < 0.01), DFA_2__ap was significantly lower for the ECHB than EO (*p* < 0.01) and EC (*p* < 0.01), while DFA_1__ml (*p* < 0.01) significantly decreased as conditions became more challenging. Medium to large effect sizes were found for DFA_1__ml, DFA_1__ap, DFA_2__ml, DFA_2__ap, %DET_ap, and %LAM_ap, ranging from 0.120 to 0.172 (Table 7). No significant age by condition interaction effects for DFA_1__ml, DFA_1__ap, DFA_2__ml, DFA_2__ap, %DET_ap, and %LAM_ap was founded.

**Table 7:** Main effects of condition on nonlinear methods of standing balance.

	EO mean	EC mean	ECHB mean	F	*p*	Effect size (*η*_p_^2^)
DFA_1__ml	1.750^+^^*^	1.726^+^^#^	1.703^*^^#^	22.015	**<0.001**	0.172
DFA_1__ap	1.717^+^^*^	1.678^+^	1.662^*^	17.666	**<0.001**	0.151
DFA_2__ml	1.123^+^^*^	0.970^+^	1.029^*^	14.776	**<0.001**	0.121
DFA_2__ap	1.171^+^^*^	1.047^+^	1.008^*^	22.012	**<0.001**	0.171
%DET_ml	80.09	80.12	79.13	1.407	0.247	0.013
%DET_ap	75.67^*^	73.93^#^	70.79^*^^#^	15.038	**<0.001**	0.120
%LAM_ml	82.66	81.89	81.67	1.761	0.174	0.016
%LAM_ap	79.78^*^	78.29^#^	75.53^*^^#^	18.211	**<0.001**	0.142

EO, eyes open; EC, eyes closed; ECHB, eyes closed and head extended backwards.

+ EO ≠ EC for *p* < 0.05.

* EO ≠ ECHB for *p* < 0.05.

# EC ≠ ECHB for *p* < 0.05.Significant differences are shown in bold font.

## Discussion

The main findings are as follows: (1) 5-year-old children showed more postural sway in the AP direction than 4-year-old children; (2) 5-year-old children showed decreased variability and more intermittent in the AP direction than 3- and 4-year-old children; (3) standing balance in the ML direction was the same for 3- to 5-year-old children; (4) as the sensory conditions became more challenging, the amount and variability of postural sway increased, while intermittency decreased; and (5) traditional and nonlinear methods provide complementary information for evaluating standing balance in preschoolers. These results are discussed below.

### Five-Year-Old Children Showed Increased Postural Sway in the AP Direction

According to traditional methods, the age-related difference of standing balance in preschoolers was only found between the 4- and 5-year-old children. This difference was shown with higher Range_ap and SD_ap in the AP direction for the 5-year-old children, consistent with the previous study (Verbecque et al., 2016a). MV_ap, MV_ml, and Path were the same for 3-, 4-, and 5-year-old children, consistent with the previous study (Verbecque et al., 2016a). The mean velocity is the most common measure used to evaluate standing balance (Wachholz et al., 2020; Xiao et al., 2020); however, it ignored some critical information about the control of standing balance (Zhou et al., 2017), especially for preschoolers. Nonetheless, based on the result of the increased postural sway of the 5-year-old children, we cannot conclude that the balance performance of the 5-year-old children has declined, given that 100% of the 5-year-old children in our study completed all three sensory conditions. In comparison, 2.56–10% of younger preschoolers failed to complete the whole test. The number of children able to cope with sensory perturbations (EC or ECHB) increased with age, consistent with previous studies (Slobounov and Newell, 1994; Verbecque et al., 2016a). Therefore, compared with younger preschool children, the balance performance of the 5-year-old children improved rather than declined, which may be associated with a person’s ability to safely explore the limits of his or her base of support with an altered control strategy (Dusing, 2016; Verbecque et al., 2016a; Lobo Da Costa et al., 2019). Increased postural sway of 5-year-old children may also be viewed as a positive adaptation to physiological development to ensure that the input to the peripheral sensory receptors exceeds the threshold for detection and enhances the sensory information available to the CNS (Carpenter et al., 2010). Besides, body height and body weight may also affect children’s balance performance, as age, body height, and body weight are positively correlated (Hsu et al., 2009). However, the control of standing balance develops during childhood reaching an optimum in early adult life (Sheldon, 1963; Goble and Baweja, 2018); age-related changes in standing balance were found with higher postural sway for 8-year-old children compared with older children (Mickle et al., 2011). These results indicate that the nonlinear developmental trend of standing balance in children (Kirshenbaum et al., 2001; Verbecque et al., 2016b), and traditional measures cannot effectively reflect the age-related changes of balance performance for preschool children.

### Five-Year-Old Children Showed Decreased Variability and More Intermittent in the AP Direction

The variability of COP sway decreased for the 5-year-old children compared with 3- and 4-year-old children, as shown with higher DFA_1__ap, consistent with the previous study (Lobo Da Costa et al., 2019). Three- and four-year-old children present a more complicated COP sway than 5-year-old children (Lobo Da Costa et al., 2019; Phinyomark et al., 2020). The higher DFA values are linked to a better standing balance performance (van den Hoorn et al., 2018). Hence, it is clearly shown that DFA can evaluate postural stability and its variations due to age-related changes (Duarte and Sternad, 2008). COP sway’s regularity increased for the 5-year-old children compared with 3- and 4-year-old children, as shown by the higher %DET_ap, consistent with the previous study (Lobo Da Costa et al., 2019). Because sample entropy (SEn) was negatively related to the regularity of COP signals (Donker et al., 2007), lower SEn values and higher %DET values indicate a more regular and predictable COP sway. In the current study, 5-year-old children presented a more predictable, less random COP motion, representing better balance performance (van den Hoorn et al., 2018). However, another study found that the regularity of COP sway reflected by SEn did not show a developmental trend in 6- to 12-year-old children (Schärli et al., 2013). These results also indicate the nonlinear developmental trend of standing balance in children (Kirshenbaum et al., 2001; Verbecque et al., 2016b).

Center of pressure sway’s intermittency increased for the 5-year-old children compared with the 3- and 4-year-old children, as shown by higher %LAM_ap. The intermittent behavior of COP motion reflects an intermittent control mechanism of standing balance (Nomura et al., 2013; Dutt-Mazumder et al., 2018; Stins and Roerdink, 2018), which is manifested as the COP motion exhibiting changes in COP dynamics from fluctuating to relatively stationary (van den Hoorn et al., 2018). In fact, a decrease in the %LAM was observed with aging for the elderly compared with the young adults, and a reduction in the %LAM was also found for older fallers compared with older non-fallers (van den Hoorn et al., 2018). These results indicate that the intermittency of COP motion increases with age for preschoolers and decreases with age for older people, which may be associated with physiological changes of development or aging (van den Hoorn et al., 2018). Therefore, 5-year-old children showed decreased variability and more intermittent in the AP direction.

### Standing Balance Was the Same for 3- to 5-Year-Old Children in the ML Direction

In contrast with the age-related changes of preschoolers’ standing balance in the AP direction, no age-related changes of standing balance for traditional and nonlinear measures were found in the ML direction, which may be associated with the control mechanism of standing balance in preschoolers or the sensory conditions adopted in the present study. For example, feet together mainly increase the difficulty of standing balance in the ML direction (Kirby et al., 1987; Izquierdo-Herrera et al., 2018), while the condition of ECHB mainly perturbs the standing balance in the AP direction (Johnson and Van Emmerik, 2012). However, age-related changes in standing balance were found in the ML direction from 4-year-old children to adults (Lemos et al., 2016), and the changes in standing balance were also found in the ML direction between autism spectrum disorder (ASD) children and typically developing (TD) children (Wang et al., 2016). Moreover, standing balance is different between the AP and ML directions (Błaszczyk and Klonowski, 2001). Therefore, the control of standing balance in preschoolers changes significantly in the AP direction while remaining unaltered in the ML direction.

### Sensory Perturbation Changed the Control of Standing Balance

As the sensory conditions became more challenging, the balance performance significantly declined, as shown by higher values of all traditional measures in the AP and ML directions. These results indicated that all traditional measures could effectively reflect the condition-related changes in preschool children’s balance performance. Moreover, the control strategy of standing balance changed significantly, as shown by lower values of fractal measures (DFA_1_ and DFA_2_) in the AP and ML directions. These results indicated that less persistent COP sway at shorter time scales and more anti-persistent pattern of COP sway at longer time scales as conditions became more challenging. Also, there was no difference between EC and EO for %DET in the AP direction, consistent with the previous study (Seigle et al., 2009). Based on these results, it can be seen that eyes closure during quiet standing on a firm surface might not influence the complexity and regularity of the standing balance in preschoolers (Seigle et al., 2009). However, head extension resulted in decreased %DET in the AP direction, which indicated that ECHB, a challenging postural task for preschoolers, caused significant changes in the regularity of the standing balance in preschoolers. These results show that the %DET is not sensitive to visual deprivation for preschoolers. Moreover, no difference was observed between EC and EO for %LAM in the AP direction, consistent with the previous study. It is clearly shown that eye closure during quiet standing on a force platform might not necessarily influence the intermittent control of the standing balance in preschoolers and young adults (van den Hoorn et al., 2018). However, the head extended backward position resulted in the decrease of the %LAM in the AP direction, which indicated that ECHB, as a challenging postural task for preschoolers, caused significant changes in the intermittent control of standing balance. These results show that the %LAM is not sensitive to the visual deprivation for preschoolers. Therefore, traditional and nonlinear methods have distinct sensitivities to different sensory perturbations. The three sensory conditions applied in the present study can effectively change the standing balance and distinguish the age-related changes of standing balance in preschoolers. This testing paradigm has the same effectiveness as the extensively used four sensory conditions on firm and foam surfaces with eyes open and closed. Moreover, under this testing paradigm, the vast majority of preschoolers can complete all the sensory conditions, which is convenient for comparison with older children, young adults, and the elderly and allows for a better understanding of the age-related changes of standing balance during the whole life span (Verbecque et al., 2016a; Goble and Baweja, 2018).

The general view is that the greater the complexity, the better the balance performance, which is contrary to this study’s results. This conflicting phenomenon may be associated with the sensory conditions adopted in this study, in which the plantar somatosensory input was not perturbated. Also, the nonstationarity of the COP signals may be another impact factor. Several studies removed the fluctuations at a lower frequency using different methods (Gow et al., 2015; Zhou et al., 2017). However, the COP signals are nonstationary data (Collins and De Luca, 1993), and the control of standing balance is intermittent (van den Hoorn et al., 2018). In fact, many studies’ results are consistent with this study’s results, which is that the lower the complexity (the higher the regularity), the better the balance performance. For example, lower complexity (MSE) of raw COP signals was found for young adults compared with the elderly (Duarte and Sternad, 2008); lower complexity (SEn) was found under the condition of eyes open compared with eyes closed (Rigoldi et al., 2014); lower complexity (SEn) was also found for 5-year-old children compared with 4-year-old children (Lobo Da Costa et al., 2019). Nonetheless, to investigate the relationship between complexity and balance performance of postural sway in children, standing balance during childhood deserves further investigation.

### Both Traditional and Nonlinear Methods Provided Complementary Information

As the sensory condition challenge increased, all traditional measures and fractal measures changed significantly in the AP and ML directions. Nonetheless, no condition-related changes in standing balance for RQA measures were found in the ML direction. It is shown that these RQA measures are not sensitive to the specific sensory condition (Seigle et al., 2009). These results indicate that all traditional measures can evaluate postural stability under different sensory conditions. However, the interpretation of these traditional measures should be considered carefully. For example, the traditional measures indicate that 5-year-old children’s standing balance showed more postural sway than 4-year-old children. In contrast, nonlinear measures suggest that 5-year-old children’s standing balance showed decreased variability, better balance performance, and more intermittency compared with 4-year-old children. These seemingly conflicting results can be explained by the free energy principle (Friston, 2010; Hur et al., 2019). The free energy principle is called a minimum entropy principle; a low entropy means that the body posture is relatively predictable (Hur et al., 2019). In fact, standing still is impossible for humans since it requires excessive efforts; little control efforts are made with flexible postural sway within a specific range. In addition, more sensory inputs are removed or reduced as the sensory conditions become more challenging (EC or ECHE), while different traditional and nonlinear methods have distinct sensitivities to the different sensory perturbations. The previous study has shown that different measures of COP signal can reflect different information of standing balance control (Luo et al., 2018). Therefore, both traditional and nonlinear methods provide complementary information for evaluating age-related and condition-related changes of standing balance in preschoolers, and the interpretation of these measures should be considered together. Based on the advantages of nonlinear methods applied to even short and nonstationary data, nonlinear methods can be used as a quantitative tool to assess gait stability in patients with different balance disorders (Sylos Labini et al., 2012) and effectively discern the gait stability between toddlers, young adults, and elderly (Bisi et al., 2014). Thus, these advanced methods can also investigate the gait in preschool and primary school-aged children.

### Limitations

In this study, the lack of randomization of the three test conditions could have induced fatigue, influencing the balance performance in preschoolers. However, we did not expect the preschoolers to fatigue because all preschoolers were allowed to rest between different conditions. Each child performed only one trial in each condition for 15 s. Future research for preschool and primary school-aged children is needed to evaluate the developmental trend of standing balance in children.

## Conclusion

Although increased postural sway, 5-year-old preschool children’s balance performance improved, and their control strategy changed significantly compared with the younger preschoolers. Sensory perturbation (eye closure and/or head extension) changed preschoolers’ balance performance and control strategy. Moreover, both traditional and nonlinear methods provided complementary information on the control of standing balance in preschoolers.

## Data Availability Statement

The data presented in this study are available on request from the corresponding author.

## Ethics Statement

The studies involving human participants were reviewed and approved by Research Ethics Board of Center for Psychological Sciences at Zhejiang University. Written informed consent to participate in this study was provided by the participants’ legal guardian/next of kin.

## Author Contributions

ZH contributed to writing the original draft, revising and editing the manuscript, data collection, data analysis, statistics, and data interpretation. YY and AH contributed to the data collection and data analysis. YG contributed to revising and editing the manuscript. JW contributed to conceptualization of the study, data interpretation, and revising and editing the manuscript. All authors approved the submitted version of the manuscript.

### Conflict of Interest

The authors declare that the research was conducted in the absence of any commercial or financial relationships that could be construed as a potential conflict of interest.
